# Associations among drug acquisition and use behaviors, psychosocial attributes, and opioid-involved overdoses

**DOI:** 10.1186/s12889-024-19217-y

**Published:** 2024-06-25

**Authors:** James A. Swartz, Peipei Zhao, Ross Jacobucci, Dennis P. Watson, Mary Ellen Mackesy-Amiti, Dana Franceschini, A. David Jimenez

**Affiliations:** 1 Jane Addams College of Social Work, University of Illinois Chicago, 1040 W. Harrison St. MC (309), Chicago, IL 60612 United States; 2 University of Notre Dame, 390 N. Corbett Family Hall, South Bend, IN 46556 United States; 3 Lighthouse Institute, Chestnut Health Systems, 221 W Walton St, Chicago, IL 60610 United States; 4 Community Outreach Intervention Projects, School of Public Health, University of Illinois Chicago, 1603 W. Taylor St, Chicago, IL 60612 United States

**Keywords:** Opioid-involved overdose, Overdose risks, SEM, Risky drug use behaviors, Risky drug acquisition behaviors, Homelessness

## Abstract

**Aims:**

This study sought to develop and assess an exploratory model of how demographic and psychosocial attributes, and drug use or acquisition behaviors interact to affect opioid-involved overdoses.

**Design:**

We conducted exploratory and confirmatory factor analysis (EFA/CFA) to identify a factor structure for ten drug acquisition and use behaviors. We then evaluated alternative structural equation models incorporating the identified factors, adding demographic and psychosocial attributes as predictors of past-year opioid overdose.

**Setting and participants:**

We used interview data collected for two studies recruiting opioid-misusing participants receiving services from a community-based syringe services program. The first investigated current attitudes toward drug-checking (*N* = 150). The second was an RCT assessing a telehealth versus in-person medical appointment for opioid use disorder treatment referral (*N* = 270).

**Measurements:**

Demographics included gender, age, race/ethnicity, education, and socioeconomic status. Psychosocial measures were homelessness, psychological distress, and trauma. Self-reported drug-related risk behaviors included using alone, having a new supplier, using opioids with benzodiazepines/alcohol, and preferring fentanyl. Past-year opioid-involved overdoses were dichotomized into experiencing none or any.

**Findings:**

The EFA/CFA revealed a two-factor structure with one factor reflecting drug acquisition and the second drug use behaviors. The selected model (CFI = .984, TLI = .981, RMSEA = .024) accounted for 13.1% of overdose probability variance. A latent variable representing psychosocial attributes was indirectly associated with an increase in past-year overdose probability (β = .234, *p* = .001), as mediated by the EFA/CFA identified latent variables: drug acquisition (β = .683,* p* < .001) and drug use (β = .567, *p* = .001). Drug use behaviors (β = .287, *p* = .04) but not drug acquisition (β = .105, *p* = .461) also had a significant, positive direct effect on past-year overdose. No demographic attributes were significant direct or indirect overdose predictors.

**Conclusions:**

Psychosocial attributes, particularly homelessness, increase the probability of an overdose through associations with risky drug acquisition and drug-using behaviors. Further research is needed to replicate these findings with populations at high-risk of an opioid-related overdose to assess generalizability and refine the metrics used to assess psychosocial characteristics.

**Supplementary Information:**

The online version contains supplementary material available at 10.1186/s12889-024-19217-y.

## Introduction

Opioid-involved overdoses and related fatalities remain persistently high in the United States and Canada despite considerable efforts to increase harm reduction interventions and treatment access [1, 2]. Per the U.S. National Center for Health Statistics, 85,145 opioid-overdose fatalities are expected for the twelve months ending in June 2023 compared with 82,325 in June 2022, a 3.4% increase [3]. Among the more prominent harm reduction efforts undertaken to reduce opioid-involved overdoses and associated fatalities are overdose education and naloxone distribution (OEND); expansion of drug checking services to test illegal drugs through point-of-care testing or by providing users test strips to detect the presence of fentanyl, xylazine, and benzodiazepines; reduction in prescription opioid availability; and the expansion of opioid agonist- and antagonist-based treatments (e.g., buprenorphine, methadone, naltrexone) for opioid use disorder [4–9].

The continuing year-over-year increases in opioid-related overdoses and fatalities have been attributed largely to the presence of fentanyl and other potent synthetic opioids in the illegal drug supply [2, 6, 10–13]. Although the fentanyl-driven increase in overdoses has largely been a North American issue to date, there is concern the use of fentanyl and other synthetic opioids such as nitazene could cause a similarly rapid increase in overdose deaths in Europe in the near future [14, 15]. Research has also identified a number of risk factors associated with higher rates of opioid-involved overdose rates. For instance, a recent study identified differences in overdose rates by biological gender, with males two to three times more likely to experience synthetic opioid-related overdoses and fatalities than females across the lifespan, even after accounting for gender-based differences in misuse and other demographics [16]. Speculating on possible reasons for the differential gender-based overdose mortality rates, the authors suggest males might have a greater propensity towards risky drug use behaviors such as “injecting alone, taking larger doses, or using untrusted suppliers”.

Additional studies and systematic reviews have identified factors other than gender and drug use behaviors that are associated with increased opioid-involved overdose and fatality risk. Some of the identified categorical risk factors include: systemic (e.g., stigma); psychosocial (e.g., unstably housed or homeless, serious mental illness, trauma exposure); demographics (e.g., age, racial/ethnic minority, and low socioeconomic status), and circumstantial (e.g., a particular dealer supplying a “hot dose”, recent release from jail or prison affecting physical tolerance, and fluctuations in potency in the local illegal opioid supply) [17–30].

For a given individual and on any particular drug-use occasion, some or all these factors likely interact in complex ways to affect overdose probability. The nature of how these factors interact to increase overdose risk, however, has largely been unexplored. Studies of multiple risk factors have typically examined them in the context of multiple regression models which, though illuminating, do not analytically consider how independent risk factors might cluster or influence each other to affect overdose probability [25, 26]. Thus, there remains a question as to why one opioid user experiences one or more unintentional overdoses over time, whereas another user experiences no or fewer overdoses. This is especially puzzling given the near ubiquity of fentanyl in the illegally manufactured opioid supply and the identification of fentanyl as a main contributing factor to drug overdoses unless, possibly, fluctuations in fentanyl potency play a larger role than currently understood [2, 31–33].

### The present study

The present study sought to develop and assess an exploratory model of how different behavioral, psychosocial, and contextual/circumstantial risk factors potentially combine to increase opioid-related overdoses. Specifically, we first assessed whether items comprising each of these domains clustered into superordinate latent factors using exploratory factor analysis (EFA). Using a second sample of participants, we next assessed this initial factor structure through confirmatory factor analysis (CFA). In the final step, we used structural equation modeling (SEM) to test whether and how the identified latent factors were associated with each other and/or with experiencing an overdose in the past year.

We hypothesized that drug use risk behaviors would directly influence the probability of an overdose and would themselves be influenced by psychosocial/demographic factors such as homelessness and gender. We also hypothesized that psychosocial/demographic factors would directly as well as indirectly affect the probability of an overdose, mediated by drug use risk behaviors.

## Methods

### Setting

We used survey interview data collected for two research projects, both of which independently recruited participants from two community-based syringe services program (SSP) sites and the surrounding communities. The sites are located on Chicago’s west and northwest sides in socioeconomically disadvantaged neighborhoods composed largely but not exclusively of racial and ethnic minorities. Among Chicago communities, both neighborhoods have relatively high opioid-involved overdose and fatality rates [34]. The SSP sites predominantly serve clients who use opioids as well as other illegally manufactured and sold drugs (e.g., cocaine, benzodiazepines, MDMA). Services provided include but are not limited to syringe exchange, naloxone and fentanyl test strip distribution, drug checking, condom distribution, and medical consultation.

Additional details on both study protocols are available elsewhere [35–37]. The first study had two components: assessing street-drug users’ current attitudes toward drug-checking services and testing the feasibility of using ecological momentary assessment (EMA) to collect data. This study’s protocol was reviewed and approved by the UIC and University of Notre Dame IRBs. The second study was a randomized controlled trial (RCT) testing the relative effectiveness of an initial telehealth versus in-person medical appointment on initiation and engagement in medication for opioid use disorder (MOUD) treatment. The UIC and Chestnut Health Systems IRBs reviewed and approved the RCT study protocol (NCT04575324). Participants in both studies provided written informed consent and were compensated with gift cards for their time and participation.

### Participants

#### EFA sample

We used the combined samples from the study on current attitudes towards drug-checking (*N* = 130) and the feasibility of using EMA to collect drug-checking data from a non-overlapping group of participants (*N* = 20) to develop a preliminary model of the factor structure of specific behaviors deemed to increase overdose risk. Both study components used convenience sampling to recruit opioid-using participants at the SSP sites between August 2021 and June 2022. Current opioid use was determined by requiring that all participants provide a small test sample of the drugs they believed to be heroin or another opioid and which they had purchased from an illegal source on the day of recruitment. The provided samples were subsequently tested off-site to assess the actual drug mixtures [38]. All participants were also required to provide information on behaviors increasing overdose risk. However, demographic information was not available for twenty participants who opted out of taking a longer survey that included the demographic questions and which was administered to the group participating in the attitudes survey component [36].

#### CFA sample

The sample used for confirmatory CFA and SEM modeling (*N* = 274) was obtained from the RCT study. Convenience sampling was also used for this study, with participant recruitment running from August 2020 through July 2022. We recruited participants from among those seeking services at the SSP sites (*N* = 92 or 33.6%) as well as by conducting street outreach in the surrounding communities to recruit current opioid users interested in beginning MOUD treatment (*N* = 182 or 66.4%). Participants were screened for an OUD at any severity level using the DSM-5 checklist of diagnostic criteria for an opioid use disorder [39]. All of the 274 participants met DSM-5 OUD diagnostic criteria, with 269 (98.1%) meeting criteria for a severe OUD. Owing to missing data on covariates used for the SEM structural analysis step, we excluded four participants (1.4%), yielding a final analytic sample of 270.

### Measures

#### Drug use behaviors

To the best of our knowledge, there is no existing validated inventory of specific drug use behaviors that increase overdose risk. We used data collected from the list of drug use behavior questions asked of participants in both studies, developed through a review of the research on drug-use overdose risk behaviors and in consultation with SSP program staff. All of the drug use behaviors as well as other measures described below were collected at baseline for participants in the telehealth study and at the initial and only interview for participants in the drug checking study. The ten specific items were: used in a new location; used with new people; used alone; had a new source/supplier; used a different (more or less) dosage than usual; used multiple drugs; used opioids with benzodiazepine; used opioids with alcohol; used for the first time in a while; and used a different administration route (e.g., injecting instead of snorting). Participants were presented with this item list and asked to select all that applied to their drug use in the past month. We dichotomized the responses into ten variables by scoring selected risk behaviors as one (1) and those unselected as zero (0).

#### Psychosocial factors

Using the CFA/SEM RCT study sample**,** we assessed participants for the following psychosocial factors potentially associated with an increased risk for an opioid-related overdose, all of which were coded dichotomously as present (1) or absent (0): severe psychological distress (SPD); homelessness; trauma exposure; and criminal justice involvement.

We assessed for SPD using Kessler’s K6-scale, a brief, widely used and validated 6-item scale consisting of questions related to symptoms of anxiety and depression (e.g., *How often in the past 30 days did you feel nervous?; How often did you feel hopeless?, *etc*.*). Scores range from 0 – 24 with scores above 12 indicating SPD and at least moderate functional impairment [40, 41].

Homelessness was assessed with a single question: *Thinking about the past year, what kind of place would you say you lived in most of the time throughout the year?* Participants who responded they were homeless and lived on the streets most of the time in the past year were counted as experiencing homelessness (1), whereas all others were scored as not homeless (0).

We assessed trauma exposure using two questions: 1) *Have you ever experienced violence or trauma in any setting (including community or school violence, domestic violence, *etc*.)* and 2) *In the past 30 days, how often have you been hit, kicked, slapped, or otherwise physically hurt?* Participants answering yes to both or either question were indicated as having been trauma exposed (1). Those answering no to both questions were coded as non-exposed to trauma (0).

To assess past-year criminal justice involvement, we used responses to the following single question, with those answering yes counted as having criminal justice involvement (1) and those responding no as not criminal justice involved (0): *Not counting minor traffic violations, were you arrested, booked, or charged for breaking a law in the past 12 months?*

#### Fentanyl preference and drug injection use

As mentioned, a considerable body of research indicates opioid-involved overdoses and fatalities occur owing to fentanyl and other synthetic opioids such as carfentanil mixed with or supplanting heroin and other drugs such as cocaine in the illegally manufactured drug supply [42–45]. Because such a large proportion of our sample had been exposed to fentanyl — baseline urinalysis testing of 240 study participants revealed a majority (*N* = 225, 93.7%) tested positive for recent fentanyl use — there was not enough variation in fentanyl use to include it as a variable. Instead, to differentiate fentanyl use among participants, we asked how many days in the past month they *intentionally* used fentanyl, reasoning that those intentionally using fentanyl would be more likely to seek out suppliers thought to provide fentanyl and/or to obtain and use illegally manufactured drugs with higher fentanyl potency. We coded those who said they intentionally used fentanyl on one or more days in the past month as having a fentanyl preference (1) and those who reported no intentional fentanyl use as having no preference for fentanyl (0).

We asked participants at baseline if they had ever injected drugs in their lifetime. Those who responded affirmatively were subsequently presented with a list of five specific drugs as well as an option to indicate any drug not listed and asked which drug or drugs they had injected in the past year. Participants who indicated injecting any drug in the past year were counted as being past-year injection drug users (1); those indicating they had never injected drugs or had not injected in the past year were counted as non-injectors (0).

#### Past-year overdose

The dependent variable in the SEM structural models was any past-year overdose. Participants were first asked: *As of today, how many times in your life would you say you have ever overdosed on drugs?* Those who responded they had overdosed one or more times were asked for their most recent overdose and the number of times in the past year they had overdosed. Because this variable was highly right-skewed and kurtotic (skew = 6.43; kurtosis = 64.77), we created a dichotomous indicator representing any past-year overdose (1) versus no past-year overdose (0).

#### Demographics

We obtained the following demographic information from participants at baseline: biological sex at birth (male or female); race (Black/African American; White, Other, Multi-racial); ethnicity (Latino/not Latino); and age in years. Race and ethnicity were coded into a single dichotomous variable represented as non-white (1) and white (0).

### Analyses

We used Stata v.17.1 [46] to generate bivariate descriptive statistics and Mplus v.8.8 for the EFA, CFA, and SEM analyses [47]. A series of bivariate statistics for both samples were generated, excluding 20 of the 150 EFA sample participants who completed a shortened interview that did not include demographic information. Significant differences were calculated within each sample, disaggregated by past-year overdose status (yes/no). Fisher’s Exact Test was used to assess statistical differences among the nominal measures and t-tests for interval/ratio level measures. For the EFA, CFA, and SEM models we used a standard set of fit statistics and thresholds to assess the adequacy of model fit: comparative fit index (CFI) >  = 0.95; Tucker-Lewis index (TLI) >  = 0.95; and root mean square error of approximation (RMSEA) <  = 0.06) [48–50].

An EFA was run using data from ten drug use risk items collected from the first sample as factor indicators. We used principal component extraction estimated by mean and variance-adjusted weighted least squares (WLSMV) to obtain the optimal factor structure based on chi-square comparisons of model fit and retaining factors with eigenvalues > 1.0. Factor extraction was followed by geomin rotation to obtain loadings for each indicator. We next ran a CFA using the RCT-based sample, constraining the model based on the best-fitting factor structure from the EFA run on the first sample. The CFA analysis on the RCT sample also used WLSMV estimation. These analyses yielded a two-factor solution (see results). As one of the factors comprised items related to use of multiple drugs and mixing specific other drugs such as benzodiazepines and alcohol with opioids, we added fentanyl preference to this factor and reran the CFA model using the RCT sample to confirm the model continued to fit the data well.

We then assessed a series of SEM structural models. We began by including psychosocial and demographic variables as well as the two latent drug use risk factors identified in the EFA/CFA steps. All SEM models included the dichotomous indicator of past-year overdose as an endogenous variable. Our intent was to identify the best-fitting, most parsimonious model that significantly predicted past-year overdose.

The first model (model 1) included all the psychosocial and demographic variables enumerated above and the two identified latent risk factors. This model included the psychosocial and demographic variables as individual exogenous predictors of any past-year overdose and incorporated direct as well as indirect pathways mediated by the latent risk factors. For the second model (model 2), we removed the direct pathways from the psychosocial and demographic factors as none were significant and included only the indirect pathways mediated by the latent factors. In the next model (model 3), we reduced the number of psychosocial and demographic indicators by removing those that did not have a significant mediated path to past-year overdoses. For the fourth model (model 4) we removed the remaining demographic variables and restructured the psychosocial variables by modeling them as a single exogenous latent factor that directly affected the two EFA/CFA-identified risk factors. This model included a direct path from the psychosocial latent factor to past-year overdose as well as paths to the drug-related latent factors to capture indirect effects on past-year overdose. In the final model (model 5), we removed the non-significant direct path between the psychosocial risk factors and past-year overdose. The specific paths and variables included in each of these models, associated model fit statistics, and the correlation matrix for the items analyzed for the final model are provided as supplementary material.

## Results

### Descriptive statistics

Table 1 shows the demographic, psychosocial, and drug use behaviors, including past year overdoses, for both study samples. Intrasample statistical comparisons between those reporting and those not reporting a past-year overdose yielded significant differences. For the EFA sample, those not experiencing a past-year overdose had experienced fewer lifetime overdoses (mean = 3.3, sd = 4.6) than those experiencing an overdose (mean = 5.8, sd = 4.9), and were less likely to report using drugs in a new location (46.2% versus 67.3%) or to have used drugs in combination with alcohol (19.2% versus 42.2%).

**Table 1:**
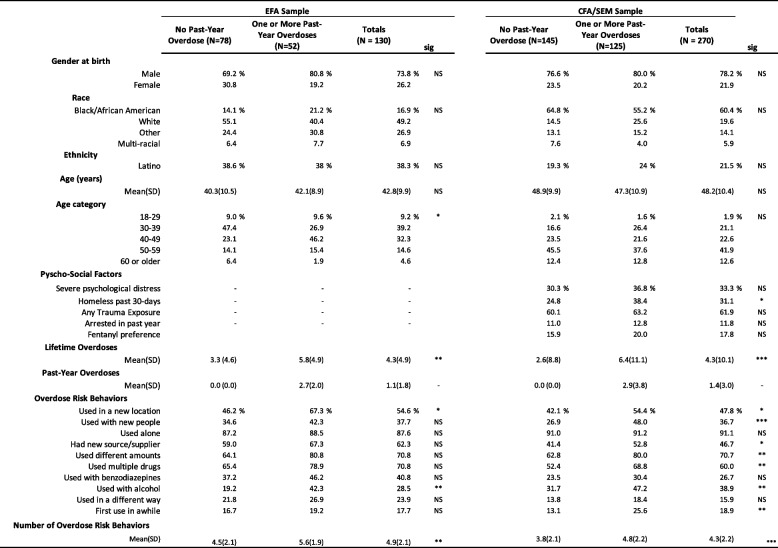
Demographics, psycho-social syndemic factors, and drug use risk behaviors by analysis sample

All significance test were calculated using Fisher's Exact Test with one-sided probabilities. Interval or ratio-level variable mean differences were assessed for significance using t-tests. The N for the EFA sample data shown above was 130. Twenty additional participants in the EFA sample who did not have demographic, syndemic, or number of lifetime or past-year overdoses, provided overdose risk behavior information, increasing the analytic N to 150 for the EFA analyses

*NS *non-significant, * = *p* < .05, ** = *p* < .01, *** = *p* < .001, - = not estimated

### Exploratory and confirmatory factor analyses 

Table 2 shows the results for the EFA and CFA measurement model. Based on data from the first sample, EFA yielded a 2-factor model as optimal based on fit statistics and scree plot analysis. A chi-square test comparing the 1- and 2-factor models was statistically significant (chi-square = 44.3, *p* < 0.001) indicating the 2-factor model afforded a better fit to the data. The 3-factor model failed to converge, suggesting it was too complex for the data available and hence, we did not consider a 4-factor model. Factor loadings for the rotated 2-factor model solution indicate the first factor is represented by items related to drug acquisition and context: using in a new location, using with new people, and having a new source/supplier. Items loading more highly on factor 2 reflected drug use risk behaviors and, in particular, polydrug use such as using alcohol and/or benzodiazepines concurrently with opioids as well as using differing amounts, using alone, and using in a different way than usual.

**Table 2:**
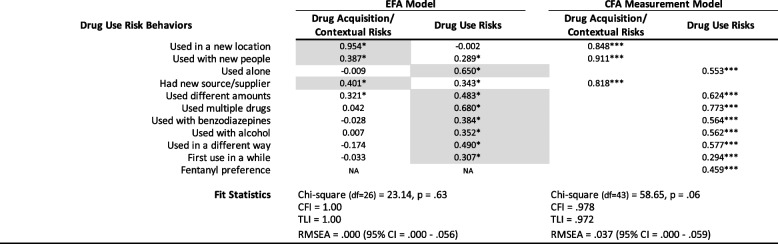
Exploratory factor analysis rotated factor loadings and confirmatory factor analysis standardized parameter estimates

EFA model sample N = 270. EFA conducted with principal components factor extraction and geomin rotation. Items shaded in grey indicate those selected as representative of a given factor in the CFA model. Parameter estimates for the CFA measurements model are standardized coefficients

*NA* not assessed, * = *p *< .05, *** =* p *< .001

The CFA results, based on model 2 and obtained by constraining the measurement model to two factors identified by the highest loading items determined by the EFA had a good fit: CFI = 0.972; TLI = 0.963; RMSEA = 0.046 (95% CI = 0.020-0.069). Because the highest loading items on factor 2 pertained to aspects of drug use per se, we added the variable representing fentanyl preference to this factor and reran the CFA. As the resulting model continued to show a good, even slightly improved fit – CFI = 0.978; TLI = 0.972; RMSEA = 0.037 (95%CI = 0.000—0.059) – we retained fentanyl preference as an indicator for the drug use factor in the subsequent structural models.

### Structural model

**Fig. 1:**
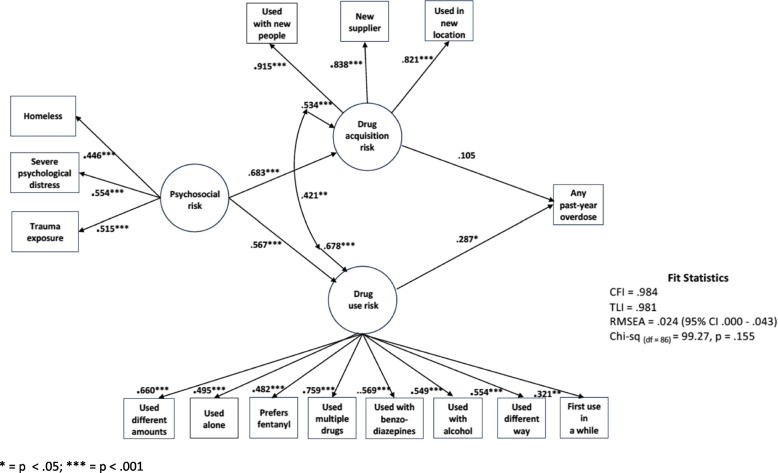
Final structural model with standardized coefficients and significant levels. * = *p *< .05; *** = *p* < .001

As indicated above, a number of alternative models were considered with details on each provided with the supplementary materials. Here, we focus on the final model as presented in Fig. 1, which shows the standardized parameter estimates; unstandardized estimates are included with the supplementary materials. This model, in our estimation, best represented the data in terms of both fit (CFI = 0.984, TLI = 0.981, RMSEA = 0.024, Chi-square _(df=86)_ = 99.27, *p* = 0.155) and parsimony. In this model, the latent variable representing psychosocial risk factors was associated with increases in drug acquisition/contextual risks (β = 0.683, *p* = 0.001) as well as risky drug use behaviors (β = 0.567, *p* = 0.001). As mediated by both latent factors, psychosocial risks were associated with an increase in the probability of a past-year overdose reflected in the total indirect effect (β = 0.234, *p* = 0.001). While there was a statistically significant association between drug acquisition and drug use risk behaviors (*r* = 0.421, *p* < 0.001), only drug use risk behaviors (β = 0.287, *p* = 0.04) but not drug acquisition/context (β = 0.105, *p* = 0.461) had a significant and positive direct association with any past-year overdose. In total, the model accounted for thirteen percent (r-square = 0.131, *p *= 0.02) of the variance in the probability of experiencing a past-year overdose.

## Discussion

### Principal findings

Revisiting study hypotheses, the results supported that drug use risk behaviors are influenced by psychosocial factors such as homelessness and that drug use risks directly influence the probability of an overdose. The results also supported the hypothesis that psychosocial risk factors indirectly affect the probability of an overdose, mediated by drug use risk behaviors. Conversely, we did not find that any demographic measure influenced drug use risk or acquisition, nor were any associated with experiencing an overdose; our assessed models fit the data well without including demographic measures. When such measures were included, there was often a decrement in model fit among the alternative models considered. We did not have specific hypotheses about the factor structure of the drug use risk behaviors considered, but the resulting two-factor structure whereby risk items relating to how drugs are acquired tended to cluster on one factor and risk items relating specifically to how drugs are used clustering on a second made intuitive sense and also fit the data well when reassessed using CFA.

We also found that participants who experienced homelessness, which was associated with experiencing severe psychological distress and trauma exposure, were more likely to engage in polydrug use, to use different amounts across occasions, and to use alone. Even absent a higher likelihood of obtaining a greater-than-expected-potency fentanyl dose, using opioids with benzodiazepines or other depressants such as alcohol also increases the risk of an overdose [51]. In our model, such drug use risk behaviors had a direct and positive association with experiencing an overdose. Riskier drug acquisition and drug use behaviors among those with psychosocial risks combined to further increase the likelihood of an overdose beyond that resulting from riskier drug use and acquisition behaviors alone.

### Strengths and weaknesses in relation to prior studies

A prior study of non-fatal overdose risks among opioid users seeking treatment also found that those with “markers of socio-structural marginalization” had a higher overdose risk [25]. Our results replicate this finding and suggest a mechanism for how being socially marginalized increases overdose risk. While we did not find a direct association between psychosocial risk and experiencing an overdose, we did find an indirect association. In our study, being homeless, having serious psychological distress, and trauma exposure, all of which were associated with each other, were associated with an increased probability an opioid misuser will engage in riskier acquisitory/contextual behaviors such as buying drugs from a new dealer or using with new people in a new location. Psychosocial risks were also associated with riskier drug use behavior such as combining opioids with benzodiazepines or alcohol, using alone, or using in a different way than usual. Our model suggests that psychosocial factors related to social marginalization are associated with an increased probability of an overdose through their association with riskier drug use and acquisition behaviors.

We might speculate that the joint effect of the psychosocial risks considered as a latent factor represents instability, the effect of which is to narrow a person’s options for consistently obtaining illegal opioids from a single supplier and for using with the same group of people in the same place. Homeless persons, who are often involuntarily displaced, are more itinerant than persons with stable housing and consequently are more likely, when acquiring and using drugs, to be among unfamiliar persons and to use in different locations [52]. Increased social and environmental instability at a time when local fentanyl concentrations in drug supplies are known to temporally and geographically fluctuate [24] could make it more likely a homeless person who uses illegal opioids obtains a “hot dose”, with higher-than-expected fentanyl potency. A recent study of the frequent involuntary displacement of homeless persons who inject drugs found that such displacements contributed to an estimated increase in mortality rates of 15.6% to 24.4% [52]. Based on our findings, we expect at least some of the heightened mortality rates in this population is due to fatal overdoses.

Somewhat surprisingly, we did not find significant associations among the assessed demographic risk factors and overdose risk. This was especially unexpected given the consistent research findings of higher overdose rates among males compared with females [16]. One thought is that studies that have identified biological sex as associated with higher overdose risk have not examined sex in combination with psychosocial, drug acquisition, and specific drug use risks. It is possible that differences among males and females on the risk factors assessed in this study could account fully or partially for the gender/sex differences found in prior studies. Unfortunately, we did not have a large enough sample to conduct a multi-group analysis of the model by biological sex to fully test this assertion. Past-year injection drug use was assessed but was also found to not improve model fit when added to the drug use latent factor nor was it independently associated with experiencing an overdose. We are not sure of the reasons for this as we expected injection use to be associated with both the drug use latent risk factor as well as with experiencing an overdose. Given that the majority of our sample, about seventy five percent, were injection users, the variation in injection use might have been too limited to detect an effect.

### Limitations

As data for this study were collected in Chicago, Illinois, in the United States and from persons who use illegally manufactured and/or sold opioids and are predominantly injection users, we do not know the extent to which the results apply to other locations and populations also at risk for an opioid-involved overdose such as those misusing prescription opioids. That we used data from participants already engaged in SSP services and/or interested in beginning treatment for an OUD could further limit the generalizability of the findings to those most amenable to harm reduction and treatment services. The sample size of 270 participants for the structural model was adequate but minimal and did not afford us the ability to test more complex models with interaction terms or additional latent factors or, as mentioned, to conduct multi-group analyses. While EFA and CFA were carried out using separate samples, we assessed the measurement and structural models using the same sample derived from the RCT study. Having additional participants would have allowed us to split the sample and test measurement and structure separately.

All study measures are based on self-report and could have been subject to over or under-reporting. Owing to extreme skew, we dichotomized past-year overdose as the dependent variable, which did not allow us to consider differences among persons who experience fewer or more overdoses over a given time. We also relied on self-report of experiencing an overdose the definition of which could vary by participant. Similarly, trauma exposure was also dichotomized out of necessity given the interview questions asked. As trauma exposure can take many forms have varying intensity and duration, our operationalization was a simplification of this construct. More detailed information on trauma exposure could provide additional insight into its association with drug use, acquisition, and overdosing.

As noted by Kline, many alternative models can fit the data equally or nearly equally as well when using SEM as an analytic technique [53]. Therefore, there is some subjectivity when selecting a “best-fitting” model. Although we attempted to examine a number of alternative models before selecting the structural model we believed best represented the data, there are certainly other models that could have been conceived of and tested and which might have been stronger statistically and/or conceptually. To remedy these limitations, other investigators might consider testing not only the model we developed but also alternative models with other data sets and samples.

Finally, our model explained only about thirteen percent of the variance in the probability of experiencing an overdose. This indicates other factors not included in the model are associated with overdose risk. These might include rapid changes in the local drug market whereby illegal drugs sold as heroin or “dope” could contain a higher dose of fentanyl or potent semisynthetic drugs than usual, or a novel drug just introduced into the local supply [24]. Then too, we asked participants to characterize their drug use and acquisition behaviors over the past three months. It is very likely that these behaviors vary from one drug use occasion to the next and that someone who is cautious one time, might be less so the next and consequently their overdose risk profile varies over time, variance for which the measurements used in this study can’t account. To capture such variance, a more dynamic model of data collection such as ecological momentary assessment would be required. [37].

### Unanswered questions and future research

To the best of our knowledge, this study represents one of the first attempts to assess the associations among opioid overdose risks across multiple functional domains: drug acquisition, drug use, and psychosocial factors. The results show these domains interact with persons who experience homelessness, have severe psychological distress, and who are trauma-exposed more likely to experience an overdose. We have speculated that the likely mechanism underlying the association between unstable social and living circumstances is that they drive acquiring and using drugs from unfamiliar sources and using in unfamiliar circumstances. Such individuals also appear to be more prone toward risky drug use behaviors, particularly polydrug use, that place them at higher risk of an overdose. We consider the results of this study to be preliminary given the limitations noted above. Research that replicates and/or extends this study’s design and methods to assess the generalizability of the model to other opioid-using populations at risk for overdosing and which includes more detailed information on complex psychosocial constructs such as trauma exposure is clearly warranted. Direct assessment of the associations among psychosocial instability and drug use risk behaviors would also be beneficial in better understanding the associative and causal mechanisms by which these factors interact to increase overdose risk.

### Supplementary Information

Supplementary Material 1.

Supplementary Material 2.

Supplementary Material 3.

## Data Availability

The datasets generated and/or analyzed during the current study are not publicly available as they contain identifying and private health information. A de-identified version is available from the corresponding author on reasonable request.
