# Supplementary material for: Associations among drug acquisition and use behaviors, psychosocial attributes, and opioid-involved overdoses
Source: BMC Public Health. 2024 Jun 25;24:1692. doi: 10.1186/s12889-024-19217-y (PMC11197316; doi:10.1186/s12889-024-19217-y)
Supplement: Supplementary file 1 — Supplementary Material 1. [file 12889_2024_19217_MOESM1_ESM.pdf]

Supplemental Table 1. Alternate SEM Models of Overdose Risk: Descriptions and Fit Statistics

Description	Psychosocial/Demographic Variables Included	Free Parameters	Fit Statistics			
			Chi-Square Test of Model Fit	CFI	TLI	RMSEA
Demographic and psychosocial variables as independent predictors of past-year overdose, with both direct and indirect effects mediated by both drug-related latent risk factors; direct effects of both drug-related latent factors included.	Age, Education, Marital Status, Gender, Racial/Ethnic Minority Status, Past-Year Injection Use, Homelessness, Serious Mental Illness, Trauma Exposure	53	148.663 (df=133, p = .17)	0.975	0.967	.021 (95% CI = .000 - .037)
Demographic and psychosocial variables as independent predictors of past-year overdose, with indirect effects only, mediated by both drug-related latent risk factors; direct effects of both drug-related latent factors included.	Age, Education, Marital Status, Gender, Racial/Ethnic Minority Status, Past-Year Injection Use, Homelessness, Serious Mental Illness, Trauma Exposure	46	156.594 (df = 132, p = .07)	0.961	0.952	.026 (95%CI = .000 - .041)
Reduced set of demographic and psychosocial variables as independent predictors with indirect effects only, mediated by drug use latent risk factor; direct effect of drug acquistion latent factor removed.	Age, Homelessness, Serious Mental Illness, Trauma Exposure	34	130.388 (df = 96, p = .01)	0.945	0.935	.036 (95%CI = .018 - .051)
Psychosocial variables combined into a single latent factor with a direct effect and indirect effects on overdose, mediated by both drug use latent factors; direct effects from both drug-related factors on overdose.	Homelessness, Serious Mental Illness, Trauma Exposure	35	99.438 (df = 85, p = .14)	0.983	0.979	.025 (95%CI = .000 - .044)
Psychosocial variables combined into a single latent factor with indirect effects on overdose, mediated by both drug use latent factors but no direct effect; direct effects from both drug-related factors on overdose.	Homelessness, Serious Mental Illness, Trauma Exposure	41	99.27 (df = 100, p = .16)	0.984	0.981	.024 (95% CI = .000 - .043)

Note. CFI = comparative fit index ; TLI = Tucker-Lewis index ; RMSEA = root mean square error of approximation ; The sample N for all models was 270.
