# Supplementary material for: Associations among drug acquisition and use behaviors, psychosocial attributes, and opioid-involved overdoses
Source: BMC Public Health. 2024 Jun 25;24:1692. doi: 10.1186/s12889-024-19217-y (PMC11197316; doi:10.1186/s12889-024-19217-y)
Supplement: Supplementary file 2 — Supplementary Material 2. [file 12889_2024_19217_MOESM2_ESM.pdf]

Supplemental Table 2. Zero Order Correlations Among Modeled Variables

	1	2	3	4	5	6	7	8	9	10	11	12	13
**Demographics**													
**Psychosocial Factors**													
1. Trauma exposure	-												
2. Housing	0.167	-											
3. Serious psychological distress	0.305	0.277	-										
**Drug Acquistion/Contextual Risks**													
4. Used with new people	0.298	0.351	0.263	-									
5. New supplier	0.341	0.280	0.277	0.691	-								
6. Used in new location	0.293	0.307	0.277	0.794	0.710	-							
**Drug Use Risks**													
7. Used different amounts	0.223	0.142	0.379	0.380	0.528	0.394	-						
8. Used alone	0.089	-0.097	0.000	0.249	0.375	0.203	0.410	-					
9. Prefers fentanyl	0.284	0.120	0.294	0.302	0.199	0.180	0.318	0.384	-				
10. Used multiple drugs	0.143	0.126	0.265	0.445	0.319	0.368	0.333	0.420	0.313	-			
11. Used with benzodiazepines	0.235	0.113	0.267	0.304	0.263	0.213	0.206	0.183	0.358	0.623	-		
12. Used with alcohol	0.139	0.063	0.053	0.428	0.356	0.189	0.248	0.278	0.157	0.561	0.173	-	
13. Used different way	0.096	0.027	0.188	0.316	0.316	0.284	0.439	0.469	0.278	0.296	0.392	0.279	-
14. First use in awhile	0.195	0.081	0.074	0.187	0.110	0.193	0.120	0.255	0.192	0.196	-0.025	0.146	0.378
