# Supplementary material for: Associations among drug acquisition and use behaviors, psychosocial attributes, and opioid-involved overdoses
Source: BMC Public Health. 2024 Jun 25;24:1692. doi: 10.1186/s12889-024-19217-y (PMC11197316; doi:10.1186/s12889-024-19217-y)
Supplement: Supplementary file 3 — Supplementary Material 3. [file 12889_2024_19217_MOESM3_ESM.pdf]

Supplemental Table 3. Unstandardized Parameter Estimates and R-square Results for the Selected SEM Structural Model

	Estimate	S.E.	Sig.	R-square	Sig
**Drug Acquisition Risk by:**					
Used with new people	1.000	0.000	-	0.837	***
Used in a new location	0.921	0.072	***	0.711	***
Had new source/supplier	0.895	0.067	***	0.670	***
**Drug Use Risk by:**					
Used different amounts	1.000	0.000	-	0.435	***
Used alone	0.750	0.182	***	0.245	*
Used multiple drugs	1.151	0.163	***	0.577	***
Used with benzodiazepines	0.862	0.162	***	0.324	**
Used with alcohol	0.832	0.142	***	0.301	***
Used in a different way	0.840	0.168	***	0.307	***
First use in a while	0.487	0.157	***	0.103	NS
Fentanyl preference	0.730	0.174	***	0.232	*
**Psychosocial Risk by:**					
Trauma exposure	1.000	0.000	-	0.266	*
Homelessness	0.865	0.268	***	0.199	**
Serious psychological distress	1.075	0.299	***	0.307	*
**Drug Acquisition Risk on Psychosocial RIsk**	1.212	0.348	***	-	
**Drug Use Risk on Psychosocial Risk**	0.726	0.24	**	-	
**Past-Year Overdose on:**					
Drug acquisition risk	0.114	0.155	NS	-	
Drug use risk	0.434	0.218	*	-	
**Drug Acquisition Risk with Drug Use Risk**	0.153	0.079	NS	-	
**Total Indirect Effect of Psychosocial Risk on Past-Year Overdos**	0.454	0.176	**	-	
**Any Past-Year Overdose**	-	-		0.131	*

Note. Figures shown are unstandardized parameter estimates, standard errors, and the r-square statistic based on the final SEM structural model.

- = Not estimated; NS = Non-significant; * = < .05; ** = < .01; *** = < .001.
